# Insight into the Life Cycle of Enterovirus-A71

**DOI:** 10.3390/v17020181

**Published:** 2025-01-27

**Authors:** Qi Liu, Jian-Er Long

**Affiliations:** 1 Key Laboratory of Medical Molecular Virology (MOE/NHC/CAMS), School of Basic Medical Sciences, Shanghai Medical College, Fudan University, Shanghai 200032, China; 23211010050@m.fudan.edu.cn; 2 Department of Pathogenic Biology, School of Basic Medical Sciences, Shanghai Medical College, Fudan University, Shanghai 200032, China

**Keywords:** enterovirus 71, life cycle, virus–host interaction

## Abstract

Human enterovirus 71 (EV-A71), a member of the *Picornaviridae* family, is predominantly associated with hand, foot, and mouth disease in infants and young children. Additionally, EV-A71 can cause severe neurological complications, including aseptic meningitis, brainstem encephalitis, and fatalities. The molecular mechanisms underlying these symptoms are complex and involve the viral tissue tropism, evasion from the host immune responses, induction of the programmed cell death, and cytokine storms. This review article delves into the EV-A71 life cycle, with a particular emphasis on recent advancements in understanding the virion structure, tissue tropism, and the interplay between the virus and host regulatory networks during replication. The comprehensive review is expected to contribute to our understanding of EV-A71 pathogenesis and inform the development of antiviral therapies and vaccines.

## 1. Introduction

Enterovirus 71 (EV-A71), classified within the Enterovirus species A of the *Picornaviridae* family, is defined by its positive-sense single-stranded RNA genome. Primarily recognized as a causative agent of hand, foot, and mouth disease (HFMD), EV-A71 shows a particular propensity for infecting infants aged 1–3 years. In severe cases, the virus can lead to neurological complications such as aseptic meningitis and brainstem encephalitis, which may result in fatal outcomes [1,2]. Although rare, adult infections with EV-A71 have been reported, with clinical presentations including fever and herpetic eruptions on the hands, feet, and oral mucosa [3,4].

EV-A71 was first identified in the Netherlands in 1963, marking an early record of its prevalence [5]. The virus was later isolated in 1969 from an infant suffering from encephalitis in the United States. Since these initial reports, EV-A71 has emerged as a significant global pathogen, with a pronounced increase in cases observed in the Asia–Pacific region [5]. The incidence of HFMD, which is mainly caused by EV-A71 and other human enteroviruses, including coxsackievirus A16 (CAV16), CAV10, and CAV6, typically peaks between June and July annually [6,7]. A variety of hypotheses have been proposed to explain the seasonal fluctuations in EV-A71 infections, including variations in host immune responses, changes in population dynamics due to seasonal social behaviors, the circulation of different viral genotypes, and the influence of solar radiation intensity [8,9,10]. Following the widespread introduction of EV-A71 vaccination in China by the end of 2015, there has been a significant reduction in the prevalence of the virus within the country. However, this has been accompanied by an observed increase in the incidence of other enterovirus serotypes and a shift in the peak periods of HFMD epidemics [11].

Transmission of EV-A71 predominantly occurs through close contact and the fecal–oral route. Once the virus enters the respiratory or intestinal epithelium, it can rapidly infiltrate surrounding dendritic cells. These cells then facilitate the spread of the virus to other tissues via the bloodstream during their migration [12]. The exact portal through which EV-A71 gains access to the circulatory system remains a subject of debate. Empirical data indicate that the detection rate of the virus in throat swabs is significantly higher than that in rectal swabs or fecal matter, suggesting a potential preference for the oropharynx as a significant site for viral entry [2]. Upon infection, the squamous epithelium lining the tonsillar crypts typically exhibits pathological changes, including edema, congestion, vesicle formation, and superficial ulceration. This suggests that the epithelium of the tonsillar crypts may be an initial site of viral entry [12]. In severe cases, infections can lead to pulmonary edema and medullary destruction, triggering hyperactivation of the sympathetic nervous system and causing an excessive influx of blood into the pulmonary system [7,13,14]. Dysregulated cytokine activation often results in an exacerbated inflammatory response, which may increase pulmonary vascular permeability. This pathological process is similar to that observed in acute respiratory distress syndrome (ARDS), where an uncontrolled inflammatory cascade leads to increased permeability of the pulmonary vasculature and subsequent respiratory distress [14]. Infections can also induce brainstem encephalitis, leading to neurogenic pulmonary edema and a range of other neurological sequelae [15]. After poliovirus (PV), EV-A71 has emerged as a significant neurotropic enterovirus that urgently requires scientific inquiry. Studies using murine models have demonstrated that EV-A71 exhibits robust neurotropism and is capable of axonal retrograde transport, highlighting its potential for neuronal infection and associated pathogenesis [16].

EV-A71 is hypothesized to breach the blood–brain barrier (BBB) and enter the central nervous system (CNS) through several potential ways [17]. Initially, EV-A71 may access the CNS by infecting cerebral microvascular endothelial cells, which are integral to the BBB’s permeability and structural maintenance. Secondly, the virus could potentially propagate retrogradely along axons to reach spinal motor neurons via neuromuscular junctions. Furthermore, peripheral immune cells might act as Trojan horses, facilitating the viral crossing of the BBB [18]. EV-A71 is also believed to target peripheral motor nerves, possibly entering through skeletal muscle and then navigating to the CNS via neural pathways. The brainstem is a common site of EV-A71 infection. Autopsy studies of individuals who succumbed to EV-A71 infection have demonstrated the notably inflammatory responses in the gray matter of the spinal cord, brainstem, hypothalamus, subthalamic nucleus, and dentate nucleus [19].

**Figure 1:**
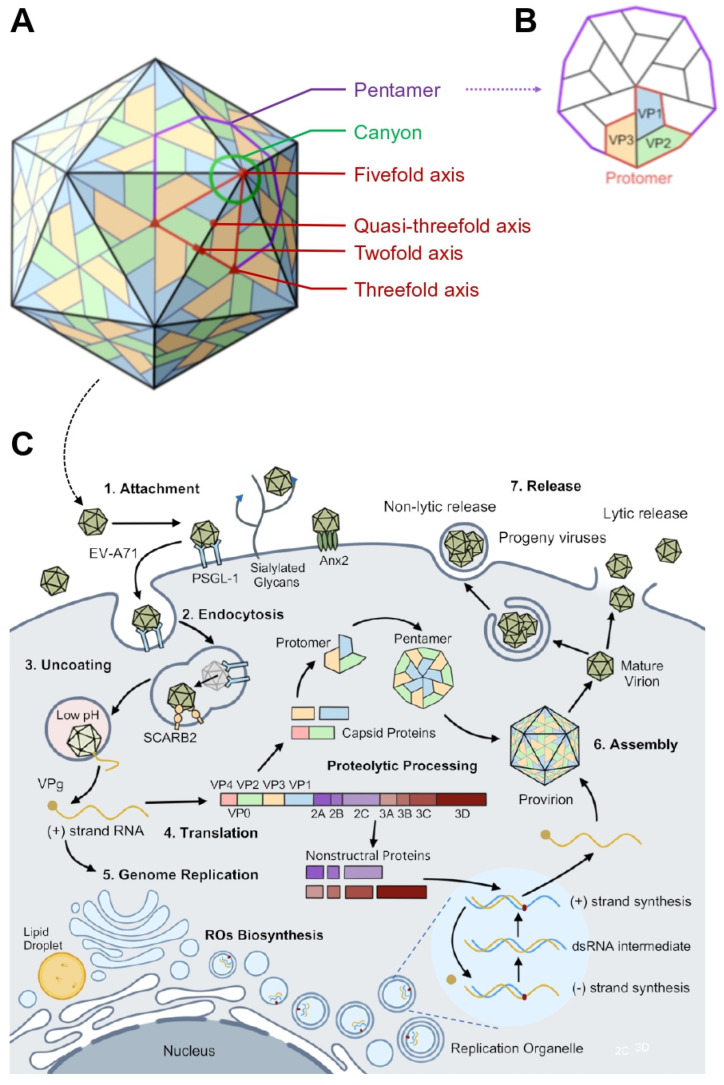
**Overview of the EV-A71 virion structure and its life cycle.** (**A**) EV-A71 virion showing the pentamer (purple line), the different symmetry axes (red point), and the location of the canyon (green circle). Twelve pentamers constitute the icosahedral capsid. (**B**) The protomer, delineated by a red line, is composed of the viral proteins VP1, VP2, VP3, and the internal VP4, with five protomers assembling into a pentameric structure. (**C**) A summary of the EV-A71 life cycle. After binding to some specific receptors and internalization, the virion releases its genomic RNA across the endosomal membrane into the cytoplasm, facilitated by the uncoating receptor and low pH. Translation of the genome results in the polyprotein that is subsequently cleaved into structural proteins (VP0, VP1, VP3) and nonstructural proteins (2A–2C and 3A–3D). For genome replication, replication organelles are derived from the Golgi apparatus and lipid droplets. The replication process, catalyzed by the 3D^pol^ enzyme, begins with the synthesis of a minus RNA strand, which serves as a template for the production of new positive RNA strands. These +RNA strands can either re-enter the replication cycle or be encapsidated into the capsids to form progeny virions. After the cleavage of VP0 into VP2 and VP4, the subsequent release of mature virions from the host cell occurs through either lytic or non-lytic mechanisms.

In this review, we would focus on the EV-A71 life cycle, with a particular emphasis on recent advancements in understanding the virion structure, tissue tropism, and the interplay between the virus and host regulatory networks during replication. The EV-A71 virion, a non-enveloped virus, is featured by its 30 nm icosahedral capsid. This capsid is composed of four structural proteins known as VP1, VP2, VP3, and the internal protein VP4, which together encapsulate the viral genomic RNA, constituting the intact virus particle. The capsid possesses distinct topographical features, including the fivefold axis, canyon region, twofold axis, threefold axis, and the quasi-threefold axis (Figure 1A). The structures are essential for virion-receptor interactions, facilitating viral attachment, uncoating, and the subsequent release of the viral genome into the host cell.

During EV-A71 infection, the “canyon region” and other key recognition sites on the virion facilitate viral uptake and decapsidation, leading to the release of the viral genome into the cytoplasm [7,20,21]. The genomic RNA then functions as mRNA, initiating cap-independent translation mediated by the internal ribosome entry site (IRES), resulting in the synthesis of a large polyprotein. This polyprotein is subsequently cleaved by viral proteases 2A^pro^ and 3C^pro^/3CD into mature proteins. Non-structural proteins 2C/2BC participate in the assembly of replication organelles and the establishment of a lipid-rich microenvironment, optimizing the intracellular conditions for viral genome replication. Here, replication complexes are formed, and 3D^pol^, the RNA-dependent RNA polymerase, initiates viral RNA replication. The newly synthesized positive-strand RNA serves as a template for both genome replication and viral protein synthesis. The VPg-linked genomic RNA and capsid proteins are then assembled into progeny virions [7]. Progeny virion release primarily occurs through cell lysis but can also be facilitated by cellular autophagy in a non-lytic way [22,23] (Figure 1C).

## 2. EV-A71 Entry into the Cells

### 2.1. Viral Attachment

The initiation of the EV-A71 life cycle is characterized by its interaction with a variety of cell surface receptors, which facilitate viral attachment, receptor engagement, and endocytosis. During the cell entry, EV-A71 virions first adhere to the cell surface via multiple glycoproteins and bind to specific receptors, initiating viral uncoating and facilitating the formation of pores through which the viral genomic RNA is released into the host cell cytoplasm. The specific receptors of EV-A71 are critical factors contributing to the viral tissue tropism.

EV-A71 engages a variety of receptors for cell entry, including scavenger receptor class B member 2 (SCARB2), P-selectin glycoprotein ligand 1 (PSGL-1), annexin II (Anx2), dendritic cell-specific ICAM-3-grabbing nonintegrin (DC-SIGN), and sialic acid (SA) [24,25,26]. These receptors can be functionally classified into two groups: uncoating receptors and adhesion receptors. Uncoating receptors, upon virus binding, induce allosteric changes in the viral particle that promote the release of the viral genome. In contrast, adhesion receptors do not mediate allosteric transformations or genome release but rather enhance the adherence of the virus to the host cell, facilitating the initial stages of infection. This functional distinction is crucial for understanding the mechanisms of EV-A71 cell entry and its subsequent replication. Despite the established role of certain receptors in facilitating EV-A71 infection, the detailed molecular mechanisms underlying this facilitation remain incompletely elucidated. To date, only SCARB2 has been identified as the primary uncoating receptor for EV-A71. Other host cell receptors involved in EV-A71 cell entry have not been definitively associated with the uncoating process and are, as a result, tentatively classified as adhesion receptors.

#### 2.1.1. EV-A71-Uncoating Receptor

SCARB2, a type III transmembrane protein, has been established as a receptor for all tested EV-A71 strains, playing a crucial role in viral attachment, endocytosis, and uncoating. As a sialylated glycoprotein, SCARB2 can interact with EV-A71 in its sialylated, desialylated, or deglycosylated forms, with desialylation modulating the interaction [27]. This receptor is expressed in various cells, including neurons, glial cells, and epithelial cells in the gastrointestinal, respiratory, and pulmonary tracts [28]. Notably, SCARB2 expression in neurons and epithelial cells highlights its role in viral infection and subsequent neurological pathogenesis [12]. A human SCARB2 (hSCARB2)-transgenic mouse model displayed paralysis symptoms similar to those observed in humans during EV-A71 infection [29]. EV-A71 antigens were detected in the neurons of the brainstem, cerebellar nuclei, and spinal cord of these transgenic mice, suggesting that hSCARB2 alone may be sufficient to induce neurological disorders following EV-A71 infection. In another transgenic murine model where the human SCARB2 gene expression was driven by a pan-promoter, the essential role of SCARB2 in EV-A71 susceptibility was reaffirmed [30]. Although SCARB2 is ubiquitously expressed, the efficiency of viral replication varied across different tissues, suggesting that tissue-specific expression of hSCARB2 is crucial for the development of murine disorders that emulate EV-A71-infected patients.

The relationship between SCARB2 and clathrin-mediated endocytosis, a critical process for the internalization of EV-A71 into host cells, was demonstrated by Hussain et al. [31]. Inhibition of clathrin-dependent endocytosis significantly impeded viral entry, an effect not replicated by inhibitors of caveolin-dependent endocytosis or macropinocytosis. Chlorpromazine, a specific inhibitor of clathrin-mediated endocytosis, was shown to markedly inhibit EV-A71 infection, while caveolin endocytosis inhibitors did not have a comparable effect [32]. Additionally, microRNAs (miRNAs) were identified to modulate the tissue tropism of EV-A71 through regulation of SCARB2 expression. For instance, miR-127-5p targets the 3′-UTR of SCARB2, thereby repressing its expression in fibroblasts [33,34]. Conversely, a decrease in hsa-miR-3605-5p was observed to increase SCARB2 expression in HEK293T cells, enhancing their susceptibility to both EV-A71 and CVA16 [35].

However, a recent study has demonstrated that SCARB2 is not present on the surface of virus-permissive cells but is instead localized within late endocytic vesicles and lysosomes. This finding excludes the possibility of SCARB2 serving as an adhesion receptor and supports its role as an uncoating receptor, as EV-A71 replication is significantly reduced in SCARB2-knockout cells, highlighting the indispensable nature of SCARB2 in the viral infection process [36].

#### 2.1.2. EV-A71 Adhesion Receptors

PSGL-1, a cell surface glycoprotein predominantly expressed on myeloid cells, lymphocytes, dendritic cells, platelets, and macrophages within lymph nodes and intestinal mucosa, as well as nervous tissues, has been identified as one of the receptors for EV-A71 [37,38,39]. PSGL-1 ability to interact with EV-A71 is strain-specific, with only a subset of strains demonstrating binding affinity, leading to the classification of EV-A71 into PSGL-1-binding (PB) and non-binding (non-PB) strains [39]. The binding specificity is determined by the presence of glycine (Gly) or glutamic acid (Glu) at the VP1 residue 145, which influences the exposure of lysine (Lys) residues at the fivefold axis of the virion [40]. Phylogenetic analysis indicates that approximately 80% of EV-A71 isolates are non-PB strains, yet the pathogenesis differences between PB and non-PB strains remain unknown. Research emphasized the importance of PSGL-1 expression on human plasmacytoid dendritic cells (pDCs) for attachment of PB strains and initiation of viral entry, ultimately triggering pDCs to secrete IFN-α and suppress the viral infection before replication [41]. However, transgenic animal models expressing PSGL-1 alone have not been sufficient to confer pathogenicity, suggesting the complexity of EV-A71 interaction with its host [42].

PSGL-1 has been implicated as a receptor for certain strains of EV-A71. However, the infection efficiency facilitated by PSGL-1 is significantly lower compared to that mediated by SCARB2, with cytopathic effects associated with PSGL-1 becoming apparent only several days post-infection [43]. PSGL-1 is thought to facilitate viral entry via a caveolin-dependent mechanism, but the absence of detectable uncoating products in infected cells suggests that PSGL-1 may bind to and internalize the PB strain of EV-A71 without inducing the conformational changes necessary for uncoating [44]. Monoclonal antibodies specific to PSGL-1 have been shown to significantly impede EV-A71 infection in cells expressing this glycoprotein, including leukocytes, but not in cells lacking PSGL-1 expression [45]. These findings suggest that while PSGL-1 enhances the infective capacity of the PB strain, it is not considered the primary receptor for the majority of host cells and is not a pivotal factor in viral pathogenesis.

Sialic acid (SA), a key mediator of EV-A71 adhesion to host cells, is prevalent in glycoproteins and glycolipids on gastrointestinal and respiratory epithelia. SA serves as a cell surface ligand for various viral hemagglutinins (HA) and viral proteins, including those from influenza virus, parainfluenza virus, reovirus 3, adenovirus 37, human rhinovirus 87 (HRV87), human enterovirus 70 (HEV70), CVA24, and hepatitis A virus [27]. Studies have demonstrated that the interaction of EV-A71 with O-linked glycans or SA-bearing glycolipids on intestinal cells triggers viral conformational changes and enhances receptor binding [46]. Depletion of cell surface SA through neuraminidase treatment or inhibition of O-linked polysaccharide synthesis significantly reduces EV-A71 adhesion to cells [27]. These findings collectively underscore the role of cell surface SA in facilitating EV-A71 binding.

Nucleolin (NCL), a multifunctional phosphoprotein localized on the cell membrane, has been identified as a modulator of EV-A71 adsorption, independent of sialic acid and glycoprotein interactions. EV-A71 VP1 protein binds directly to non-glycosylated NCL, and suppression of NCL expression reduces viral binding to host cells. Murine cells expressing human NCL exhibit increased susceptibility to EV-A71 infection [47]. Electron microscopy analysis of RD cells infected with EV-A71 and double-stained with gold-labeled antibodies against SCARB2 and NCL revealed substantial colocalization of the two molecules, suggesting a synergistical role in EV-A71 binding and clathrin-dependent endocytosis [32,47].

Vimentin, a protein crucial for maintaining cellular morphology and cytoplasmic integrity, serves as a structural component that supports and anchors cellular organelles, including the nucleus, endoplasmic reticulum, and mitochondria. It is also implicated in the attachment and endocytosis of various viruses, potentially enhancing their adhesion to other cellular surface molecules [48]. EV-A71 VP1 protein can bind to surface vimentin, and reducing vimentin expression impairs viral attachment. Moreover, the supplementation of exogenous vimentin or the use of anti-vimentin antibodies significantly suppresses viral multiplication. However, increasing the concentration of vimentin antibodies or exogenous vimentin was not sufficient to completely block viral infection, suggesting that vimentin acts as an early cofactor in virus entry rather than being essential for the infection process itself [49].

Annexin II (Anx2), a 36 kDa calcium-binding protein from the annexin family, is predominantly expressed on endothelial cell surfaces [50,51]. The interaction between EV-A71 and Anx2 is hypothesized to occur within the amino acid residues 40–100 of the VP1 protein, which includes the canyon region. Pre-treatment of host cells with Anx2 or Anx2-specific antibodies has been shown to inhibit EV-A71 adsorption [51]. All five distinct genotypes of EV-A71 strains can directly bind to Anx2, suggesting that Anx2 may primarily function as an adhesion receptor.

Heparan sulfate and DC-SIGN have also been identified as receptors for EV-A71, facilitating viral attachment to cells [52]. Additionally, cyclophilin A (CypA) and recombinant human tryptophan-tRNA ligase (WARS) have been identified as uncoating regulators and entry mediators, respectively, conferring susceptibility to cells that are otherwise non-permissive in the absence of SCARB2 [48]. Prohibitin (PHB) is implicated in the pathogenesis of the nervous system associated with EV-A71 infection. Cell surface PHB is involved in specific recognition and EV-A71 entry into neuronal cells, while mitochondrial PHB is associated with the assembly of viral replication complexes [53].

### 2.2. Virus Internalization

Receptor-mediated endocytosis is a well-established mechanism for the internalization of macromolecules and serves as a primary route for the entry of non-enveloped viruses into host cells. EV-A71 utilizes multiple endocytic pathways for cellular entry. For instance, EV-A71 interacts with SCARB2 and enters RD cells through a clathrin-dependent endocytic mechanism [31,32], Additionally, EV-A71 engages with PSGL-1 to enter Jurkat T lymphocytes via a caveolin-dependent pathway [45]. Despite these findings, the molecular mechanisms facilitating EV-A71 penetration into neural cells remain largely elusive. Other endocytic pathways, such as micropinocytosis, which are independent of clathrin and caveolin, are employed by various viruses but have not been identified as entry routes for EV-A71.

#### 2.2.1. Clathrin-Mediated Endocytosis

Research has indicated that blocking clathrin-mediated endocytosis (CME) significantly reduces EV-A71 infection in RD cells, confirming that EV-A71 utilizes CME for cellular entry [31]. Clathrin and adaptins are key players in this endocytic process. Clathrin, known for its “tripod” triskelion structure consisting of three heavy chains and three light chains, assembles to create a lattice on the endosomal membrane. Adaptins are capable of binding simultaneously to clathrin, membrane phospholipids, and transmembrane proteins, thus coordinating the assembly of the clathrin lattice on the cell membrane. Endocytosis is initiated by the interaction of EV-A71 with host cell surface receptors, which then recruit clathrin and adaptins to form a clathrin-coated compartment. This interaction triggers the invagination of the cell membrane, leading to the formation of an endocytic pit that constricts into a vesicle, approximately 70–150 nm in diameter, which lacks the outer clathrin lattice. These vesicles are subsequently internalized into the cytoplasm [54].

#### 2.2.2. Caveolin-Mediated Endocytosis

The entry of EV-A71 into RD cells expressing SCARB2 was significantly inhibited by blockade of CME, suggesting that CME is a primary route for EV-A71 entry into these cells. In contrast, the entry of EV-A71 into Jurkat T lymphocytes and transgenic mouse L929 cells expressing PSGL-1 was largely unaffected by CME inhibitors, indicating that these cells utilize a different endocytic pathway for viral entry, likely caveolae-mediated endocytosis (CaME) [45]. CaME is a prevalent clathrin-independent endocytic mechanism characterized by the formation of endocytic vesicles, 60–80 nm in diameter, which arise from the invaginations of the cell membrane. Unlike CME, caveolins persist within the vesicles following their formation, highlighting a fundamental difference in the molecular composition and dynamics of the vesicle coat in CaME.

## 3. EV-A71 Uncoating and Genome Release

The molecular mechanisms of EV-A71 uncoating are not yet fully elucidated. However, by drawing on knowledge from other enteroviruses, it is proposed that EV-A71 uncoating initiates after receptor binding and cellular internalization. The native virion is believed to swell and undergo allosteric changes to form A-particles, which facilitate the extrusion of the viral genome. Subsequently, the viral genome is released into the cytoplasm under the influence of specific inducing factors, resulting in an empty capsid being retained within the endosome.

The five-fold axis region of EV-A71, composed of the VP1 protein, is pivotal to the uncoating process, featuring two critical structures: the “canyon” and the “pocket factor”, which are essential for virion stability (Figure 1A). The VP1 protein includes six loops (BC, DE, BE, GF, GH, HI) that are characterized by their multi-epitope nature and high conservation. Notably, three neutralizing monoclonal antibodies have been observed to bind simultaneously to a conserved epitope on the GH loop, hindering viral attachment and internalization, thus underscoring the GH loop’s role in viral entry [55]. Furthermore, a neutralizing epitope has been identified in close proximity to the GH loop, which, together with the EF loop of the VP2 protein, forms a significant epitope for viral neutralization [56]. Although various cell surface receptors have been implicated in EV-A71 infection, only SCARB2 has been shown to be capable of mediating viral uncoating, highlighting its unique role in this process.

### 3.1. SCARB2-Mediated EV-A71 Uncoating

Enteroviruses, such as poliovirus, coxsackievirus, and rhinovirus, initiate infection through interactions between immunoglobulin-like (Ig-like) structures within the canyon and their respective receptors [57]. EV-A71 also possesses a canyon proximal to the five-fold axis of its capsid. However, the canyon of EV-A71 is relatively shallow compared to other enteroviruses, potentially impeding the binding to Ig-like receptors [58]. Within the mature EV-A71 virion, a hydrophobic pocket is located at the base of the canyon, housing a “pocket factor” that may be intricately associated with virion stability. The chemical identity of the “pocket factor” remains a subject of debate. Plevka et al. [57] suggest it is lauric acid, while Wang et al. [59] propose it to be sphingosine.

EV-A71 undergoes three structural transitions during the uncoating process. Initially, the native virion (160 S) exhibits a stable configuration with high infectivity. Subsequently, the A-particle (135 S) emerges as an unstable intermediate in the uncoating cascade, retaining some degree of infectious capability. The final state, characterized by the empty capsid (80 S), is devoid of infectivity and represents the remnants of the virion at the terminal phase of uncoating [60]. While both the native virions and empty capsids are natural viral particles, the former is capable of undergoing reversible conformational alterations in different environments and transiently revealing VP4.

SCARB2, predominantly localized on the lysosomal membrane of host cells, was initially characterized by its role in the transport of β-glucocerebrosidase from the endoplasmic reticulum to lysosomes before being identified as a receptor for EV-A71 [61]. Studies have shown that SCARB2 binds to EV-A71, facilitating viral internalization and mediating viral uncoating under acidic conditions (pH < 6.0) within the late endosome [44]. Structural analyses of SCARB2 reveal distinct differences from the uncoating receptors of poliovirus, Coxsackievirus B (CVB), and other enteroviruses, notably the absence of Ig-like structures. SCARB2 features an antiparallel β-barrel structure accompanied by several short α-helices. Notably, two α-helices (α1 and α15) are positioned at the base of the β-barrel, connecting to the N- and C-termini of the transmembrane region. The head region consists of a triple helix bundle formed by α4, α5, and α7, along with two short helices (α2 and α14) and the β7 sequence. Specifically, the α4, α5, and α7 helices act as “gatekeepers”, undergoing conformational changes in response to varying pH levels. Upon binding of the GH loop of EV-A71 VP1 to the head region of SCARB2, α4 and α5 partially separate from α7 under the acidic environment within the late endosome, thereby opening an internal lipid transfer channel. This channel interacts with the hydrophobic pocket at the base of the canyon region, enabling the release of the “pocket factor” and facilitating uncoating [62]. In contrast, the lipid channel remains closed under neutral conditions, preventing interaction with the hydrophobic pocket.

The dual triggers for EV-A71 uncoating—the interaction between the GH loop of EV-A71 VP1 and SCARB2, along with endosomal acidification—act in concert to induce the collapse of the hydrophobic pocket located at the base of the canyon. This leads to the release of pocket factors and the conversion of the virion into an unstable 135 S intermediate, referred to as the A-particle. The ensuing allosteric transition promotes the detachment of the viral genome from the internal VP4 protein, which is then externalized and anchored to the host cell membrane via the N-termini of VP1. Simultaneously, a virion pore forms at the canyon’s base, adjacent to the five-fold and two-fold axes, facilitating the translocation of the viral genomic RNA into the cytoplasm and resulting in an 80 S empty capsid. It has been established that the initiation of EV-A71 uncoating is contingent upon both receptor binding and low pH, as evidenced by the significant inhibition of viral infection following treatment with endocytic vesicle acidification inhibitors [50].

### 3.2. Genome Release

The genomic release of EV-A71 has been a focus of research, with particular attention given to the locales and structural dynamics involved in pore formation on the viral capsid. In addition to the established five-fold axis, evidence suggests the existence of alternative conduits for genomic egress at the two-fold and quasi-three-fold axes (Figure 1A). However, the precise positioning of these channels remains unclear due to the limitations in electron microscopy resolution [60]. Naturally infected cells exhibit varying sensitivities to environmental conditions, which may lead to asymmetric impacts on virion integrity and consequently alter the genomic release.

Examination of the two states of the EV-A71 particle, the A-particle and the empty capsid, has revealed that A-particle capsids can engage with their genome at the two-fold axis, where a pore is formed with negatively charged residues extending to the virion exterior. This pore constricts subsequent to genomic release, with the overall dimensions of the capsid remaining relatively unchanged [63]. The spatial conformation transitions between the native virion, A-particle, and the empty capsid were less pronounced than anticipated [64], and further exploration of these modulations is constrained by the resolution of electron microscopy.

The stimulus for the ejection of the viral genome to form an empty capsid in EV-A71 remains unclear. It is hypothesized that genome release is not an immediate event following receptor binding or pH decrease. Staring et al. [65] demonstrated that phospholipase A2-XVI (PLA2G16) plays a significant role in the ejection of the viral genome, yet it is not involved in receptor binding, endosomal trafficking, or pore formation. It is speculated that PLA2G16 might interact directly with the viral capsid proteins or genomic RNA or modulate membrane fluidity, pore dimensions, and composition to create a lipid environment conducive to pore formation, thereby indirectly facilitating the extrusion of the viral genomic RNA.

In summary, SCARB2 primarily facilitates virus entry into cells via the “canyon region” adjacent to the five-fold axis of the EV-A71 capsid in a clathrin-dependent endocytic pathway. After binding to SCARB2, virions are translocated by endosomes into the cytoplasm. Within the acidic environment of late endosomes, the lipid transfer channel of SCARB2 is activated by interaction with the hydrophobic pocket at the base of the canyon, promoting the release of pocket factors and reducing virion stability, thus initiating SCARB2-mediated uncoating and genomic RNA release. Consequently, the native EV-A71 virion transforms into an unstable intermediate A-particle. The N-termini of VP1 and VP4 are then externalized and anchored to the endocytic vesicle membrane, forming a hexamer pore that facilitates the release of genomic RNA into the cytoplasm. Recent studies on the impact of thermostability mutations have provided a more detailed scenario of virus uncoating and genome release, where natural virions constrict into A1-particles upon uncoating stimuli, expel pocket factors and VP4 to form unstable intermediate A2- and A3-particles, and ultimately form a pore for the release of the viral genomic RNA, leaving behind an empty capsid [20].

## 4. Viral Protein Translation and Polyprotein Processing

**Figure 2:**
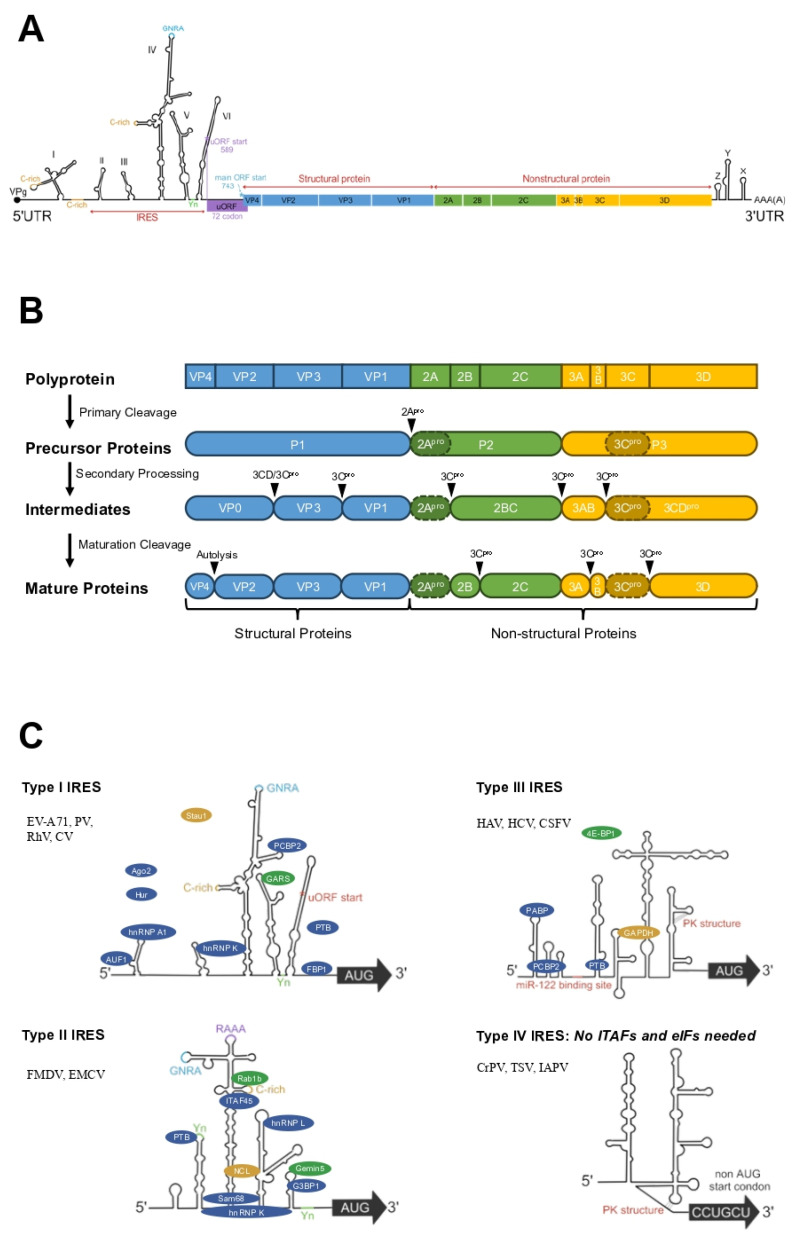
**Schematics of the EV-A71 genome, translation, and polyprotein processing.** (**A**) The EV-A71 genome and secondary structure loops (SLs) are depicted as a black line and circles, respectively. The 5′-UTR of EV-A71 encompasses SLs I–VI (black line), with SLs II–VI constituting the type I IRES. The viral major open reading frame (mORF) encodes a polyprotein precursor, which is proteolytically processed into four structural proteins (indicated with blue blocks) and seven non-structural proteins (2A–2C indicated with green, 3A–3D in yellow, respectively). Additionally, a small upstream open reading frame (uORF) is indicated by a purple block, initiated from the first in-frame AUG codon (AUG1). The 3′-UTR of EV-A71 contains domains designated as -X, -Y, and -Z, which are implicated in genome RNA replication. (**B**) A schematic illustration of the translation and processing of the EV-A71 polyprotein is presented, highlighting the primary roles of 2A^pro^ and 3C^pro^ proteases in these processes. (**C**) Four types of classical viral IRES. IRESs are generally categorized into four types based on their essential eIFs and ITAFs, secondary structure, and other biological properties. Some well-known ITAFs binding to the domain are shown. Notable domains within the IRES are color-coded: C-rich regions are in brown, representing areas with a high cytosine content; GNRA motifs are in blue, where G is for guanine, N for undefined nucleotides, R for purine (adenine or guanine), and A for adenine; Yn regions are in green, indicating pyrimidine-rich sequences approximately 8 to 10 nucleotides in length. Thin line junctions denote known pseudoknot structures within the IRES (adapted from references [21,68]).

Upon release into the cytoplasm, the EV-A71 genomic RNA is directly translated by cellular ribosomes to produce viral capsid and non-structural proteins. The viral genome is characterized by a single major open reading frame (mORF), which is bordered by a highly structured 5′-untranslated region (5′-UTR) and a 3′-untranslated region (3′-UTR) that includes a poly(A) tail (Figure 2A). Recently, a small upstream open reading frame (uORF) was identified within the EV-A71 genome, positioned between the 3′-terminus of the IRES and the N-terminus of the mORF [66,67], challenging the classic conception that enteroviruses possess only a single ORF.

The viral mORF encodes a 2193-amino acid polyprotein that is subsequently cleaved by viral proteases 2A^pro^ and 3C^pro^ into three precursor proteins, designated P1, P2, and P3. P1 is further processed to yield four structural proteins, VP1, VP2, VP3, and VP4, which constitute the viral capsid. P2 is processed into 2A^pro^, 2B, and 2C, while P3 is cleaved into 3A, 3B (VPg), 3C^pro^, and 3D^pol^ (Figure 2B). Non-structural proteins with defined roles are annotated with superscripts, such as the 2A protease (2A^pro^) and the RNA-dependent RNA polymerase (RdRp, 3D^pol^). These non-structural proteins are primarily involved in viral translation and genomic RNA replication.

The 5′-UTR of EV-A71 contains six stem-loops (SL), with SL I being crucial for genomic RNA synthesis and essential for viral replication, while SL II-VI form the viral internal ribosome entry site (IRES), directing cap-independent protein translation [68] (Figure 2A). The 3′-terminus of the IRES contains an Yn-Xm-AUG-motif (AUG1), which consists of an 8 to 10 nucleotide pyrimidine-rich region (Yn), an 18 to 20 nucleotide spacer (Xm), and an AUG start codon. This motif is proposed to serve as a ribosome entry site rather than a traditional start codon, functioning as a regulatory element that guides the translation initiation complex to the authentic initiation site (AUG2), located 30–160 nucleotides downstream, and involved in eIF1 and eIF1A-mediated mRNA scanning [69]. Studies have indicated that approximately 20% of viral protein translation may initiate at AUG1, suggesting a potential role as a translation initiation site under certain conditions, although the mechanism governing the choice between the two AUGs is not yet fully understood [70].

The 3′-UTR of EV-A71 is a highly conserved domain critical for the translation and replication of the viral genome, comprising three domains—X, Y, and Z—as well as a poly(A) tail, with domain X being the most conserved and domain Z the least conserved [71] (Figure 2A). Point mutations within the 3′-UTR can disrupt the stem-loops, thereby perturbing the 3′-UTR structure and potentially leading to a lethal phenotype [72].

### 4.1. Translation Initiation

Translation initiation for the EV-A71 polyprotein is contingent upon the assembly of the initiation complex within the viral IRES, a pivotal step in regulating viral protein translation. Most protein translation can occur via two pathways: the classical cap-dependent mechanism or the IRES-mediated cap-independent mechanism. While the majority of host protein synthesis is initiated by the cap-structure, viruses, including EV-A71, often employ diverse strategies to circumvent host translation machinery limitations due to their compact genomic coding capacity, favoring IRES-mediated translation for their replication [73]. It is noteworthy that host cell translation patterns can be altered under various stress conditions, such as nutrient deprivation, endoplasmic reticulum (ER) stress, and hypoxia, which may influence the preference for cap-dependent versus cap-independent translation initiation. Understanding these dynamics is crucial for developing strategies to modulate viral replication and pathogenesis.

#### 4.1.1. IRES-Mediated Viral Polyprotein Translation

The IRESes are generally categorized into four distinct types based on their secondary structure: required eukaryotic initiation factors (eIFs) and IRES trans-acting factors (ITAFs) (Figure 2C). Type I IRESes, exemplified by those found in PV, HRV, Coxsackievirus, and EV-A71, are characterized by six stem-loops (SLs), with SL II-VI forming the core domain for translation initiation. Type II IRESes, which consist of four SLs, show sequence and structural motifs in the 5′-UTR analogous to those of Type I and are observed in foot-and-mouth disease virus (FMDV) and encephalomyocarditis virus (EMCV). Type III IRESes are more concise and compact than Type II and are typically found in hepatitis A virus (HAV), HCV, and classical swine fever virus (CSFV). Type IV IRESes, referred to as the intergenic region (IGR) IRESes, which do not require any initiation factors and ITAFs to initiate its translation. Initially identified in cricket paralysis virus (CrPV), type IV IRES has also been observed in Taura syndrome virus (TSV) and Israeli acute paralysis virus (IAPV) [68,74]. A distinct class of IRES with eight SL motifs, identified in Aichi virus A of the *Picornaviridae*, was also classified into a new type of IRES [75].

Canonical eukaryotic mRNA translation is initiated by the 43S pre-initiation complex (PIC), which is recruited by the cap-binding complex to the 5′-terminal cap of mRNA. The eIF4E, complexed with eIF4G and eIF4A, tightly binds to the cap to form the 48S preinitiation complex, which is crucial for translation initiation. The N-terminus of eIF4G interacts with the polyadenine binding protein (PABP), which binds the poly A tail of the 3′-UTR, creating a closed loop between the mRNA 5′- and 3′-termini. This loop stabilizes the PIC, enhances translation efficiency, ribosome recycling, and re-initiation.

EV-A71, which possesses a type I IRES, orchestrates multiple eIFs to initiate polyprotein translation in a cap-independent manner. The viral 2A^pro^ and 3C^pro^ proteases cleave multiple eIFs, including eIF4G, eIF4A, eIF5B, and PABP [68]. The cleaved eIF5B then substitutes for eIF2 to deliver Met-tRNAi^Met^ to the 40S ribosomal subunit [76]. The N-terminal binding domain of eIF4G, released from cleavage by the viral 2A^pro^, significantly reduces cap-dependent translation of cellular mRNA without disrupting the eIF3-eIF4A interaction. This leads to the cessation of cap-dependent translation and indirectly promotes viral protein synthesis. For type I IRES-mediated translation, all initiation factors are required except for eIF4E, which interacts with the cellular mRNA cap [76,77]. Additionally, the central domain of eIF4G can specifically bind to SL V, recruiting eIF4A and facilitating the assembly of the 43S PIC. Some studies have observed that Death-Associated Protein 5 (DAP5) can function as a counterpart of eIF4G, capable of participating in the initiation of cap-independent translation [78].

The recently identified uORFs in EV-A71 are hypothesized to play a role in the re-initiation of the main open reading frame (mORF) translation on the same genomic RNA following the initial translation of uORFs. This re-initiation process is primarily mediated by the monocarboxylate transporter 1 (MCT-1) and the density regulatory protein (DENR), which are involved in GTP-independent Met-tRNA delivery and the initiation of uORF translation. DENR has been shown to promote translation re-initiation via ribosome recycling, which is crucial for the expression of certain oncogenes, including ATF4 [79]. Furthermore, it is deduced that, in addition to IRES-mediated translation, EV-A71-infected cells may also harbor IRES-independent translation mechanisms. This proposition is largely based on the hypothesis that viral gene recombination and protein translation are coupled, with observations indicating that the absence of IRES augments the viral genome recombination [80]. This implies a potential translation mechanism for the non-structural proteins that might facilitate viral recombination. Concurrently, it reveals the significance of non-canonical translation initiation factors, eIF2A and eIF2D, in this specific translational process [80].

#### 4.1.2. IRES Trans-Acting Factor

IRES trans-acting factors (ITAFs) are crucial host factors that are significantly involved in the cap-independent translation of EV-A71. ITAFs comprise a diverse range of molecules, including nuclear and cytoplasmic proteins and long non-coding RNAs. Their functions can be modulated by various elements, such as nuclear–cytoplasmic shuttling, viral RNA binding, post-translational modifications, and interactions with diverse eIFs. ITAFs act as molecular chaperones for IRES, capable of binding to RNA molecules across multiple domains and conferring stability to IRES structures, thereby facilitating the engagement of canonical translation factors and ribosomes [81].

The EV-A71 IRES is regulated by multiple ITAFs (Table 1), including the heterotypic nuclear ribonucleoprotein A1 (hnRNP A1), far upstream element binding protein 1 (FBP1), FBP2, and heat shock protein 27 (Hsp27). The effects of ITAFs on the translation of EV-A71 IRES are complex. For instance, FBP1, which can be cleaved by 2A^pro^, modulates the RNA binding site and enhances viral IRES activity [82]. FBP2 accumulates in the cytoplasm, where viral genome replication occurs, and interacts with IRES to inhibit viral translation [83]. Hsp27 plays a role in preventing protein aggregation during heat shock, protecting cells against stress-induced effects [84]. Hsc70, in conjunction with Hsp27, suppresses cap-dependent translation of cellular mRNA and also impedes genome replication [85]. DEAD-Box Helicase 3 (DDX3X) can bind to the SL VI of IRES, aiding in the unwinding of RNA secondary structures to facilitate ribosome entry and enhancing viral IRES-mediated, cap-independent translation [86]. The ATP-dependent RNA helicase DHX29 is crucial for EV-A71 in scanning the 5′-UTR of the viral genome, promoting the recognition of the AUG codon, and initiating translation [87,88]. It was reported that the silent mating type information regulation 2 homolog-1 (SIRT1) exerted an inhibitory effect on the EV-A71 genomic RNA replication and translation by interacting with its IRES. Conversely, the extracellular signal-regulated kinase 1/2 (ERK1/2), SRC mitogen-associated protein 68 (Sam68), and the early growth response protein 1 (EGR1) were observed to positively modulate the IRES activity [89,90,91].

### 4.2. Elongation and Termination of the Viral Polypeptide Chains

The initiation of translation is characterized by the assembly of initiation complexes, which is followed by the elongation phase and subsequently the release, folding, and processing of the polypeptide chain.

It is noticeable that miRNAs also play an important role in the regulation of viral protein translation. MiRNAs, a class of 20–25 nucleotide non-coding small RNA molecules, are implicated in the regulation of viral protein translation through diverse mechanisms. Typically, miRNAs act on their complementary mRNAs by forming the miRNA-inducing silencing complex, thereby inhibiting mRNA translation or inducing mRNA cleavage and degradation [112]. Hsa-miR-296-5p is capable of inhibiting the translation and replication of the EV-A71 BrCr strain by binding to two specific targets within the viral genome (g.2115–2135 and g.2896–2920) [113]. MiR-18a and miR-452 have been shown to directly target the VP3 RNA, reducing viral protein expressions [114]. MiRNAs can also modulate viral translation by influencing other critical translational factors. For example, miR-141 is upregulated in RD cells after EV-A71 infection, shutting off host protein translation while promoting viral protein translation through its interaction with eIF4E mRNA [115].

### 4.3. Processing of Viral Polyproteins

#### 4.3.1. Processing of Polyproteins

The translation of mORF in EV-A71 results in a polyprotein of approximately 2300 amino acids in length, which is then cleaved by the viral proteases 2A^pro^ and 3C^pro^. Initially, this polyprotein is processed into three precursor proteins: P1, P2, and P3. Further cleavage yields four structural proteins and seven non-structural proteins [60]. The primary cleavage event is a critical and rapid co-translational process. During this process, 2A^pro^ autocatalytically processes its N-terminus, separating the P1 precursor proteins responsible for capsid formation from the nonstructural P2 and P3 polyproteins. The subsequent primary cleavage occurs between 2C and 3A, facilitated by 3C^pro^ (Figure 2B). This cleavage between 2C and 3A is dependent on the prior translation of the 3C^pro^ sequence. However, it has been hypothesized that the translation of the entire downstream 3C^pro^ sequence might not be necessary, as the expression of a truncated RNA transcript does not hinder cleavage at the 2C/3A junction. Studies have also revealed the existence of a primary cleavage between 2A and 2B in PV [116], and since the 2BC and 2C products are similarly observed in the processing of the foot-and-mouth disease virus (FMDV) polyprotein [117], this indicates a similar proteolytic mechanism.

The precursor proteins resulting from the primary cleavage of the polyprotein undergo further processing into intermediate and mature proteins, a process predominantly mediated by the viral protease 3C^pro^ (Figure 2B). A single 3C^pro^ within a polyprotein is incapable of self-processing; however, multiple precursor 3C^pro^ molecules translated from the same RNA can cleave each other, thus accomplishing the secondary maturation of the precursor proteins. The proteolytic cleavage sites are characterized by their high specificity within the polyprotein, with their positioning being a principal factor in protease recognition [118]. In picornaviruses, 3C^pro^ exhibits proteolytic activity in various forms, including 3CD^pro^, 3ABC, or 3ABCD [119]. In addition, the viral 2A^pro^ of PV is also shown to target the Y-G base pair, facilitating the cleavage of 3CD proteins into 3C’ and 3D’ [120,121].

#### 4.3.2. Viral Structural Proteins

The precursor polyprotein P1 is initially cleaved into VP0 (36 kDa), VP1 (32 kDa), and VP3 (27 kDa) by the viral proteases 3C^pro^. During viral capsid assembly, VP0 is further autolyzed into the mature proteins VP2 (28 kDa) and VP4 (8 kDa), which play crucial roles in reinforcing and stabilizing the capsid structure. The capsid surface is composed of VP1, VP2, and VP3, while VP4 is positioned internally within the capsid. The cleavage of precursor VP0 between Asn and Ser residues to form VP2 and VP4 is a pivotal event in viral capsid maturation, conferring stability to the icosahedral particle [122].

In addition, VP1 possesses the primary receptor-binding epitope and includes two critical virion stabilizing structures, the “canyon” and the “pocket factor”. The release of the pocket factor, triggered by receptor binding and low pH, leads to a conformational change in the virion, which is considered a significant indicator for the discharge of the EV-A71 genome [57]. VP2 has been reported to be correlated with viral infectivity and the induction of host cell apoptosis [123]. VP3, which also includes some important epitopes for neutralizing antibodies, could serve as a potential target for diagnostic or therapeutic monoclonal antibodies (mAbs). VP4, which is internally associated with the genomic RNA within the virion, contributes to virus stability and provides another promising target for the antivirals. The other important roles of these capsid proteins are summarized in Table 2.

#### 4.3.3. Viral Non-Structural Proteins

The viral 2A^pro^ is a key protease responsible for the primary cleavage of the polyprotein translated from the viral genome. Its role extends beyond proteolytic activity, as it also regulates the extracellular signal-regulated kinase (ERK) pathway. This regulatory function of 2A^pro^ underscores its multifaceted contribution to the viral life cycle [133]. Viral 2B functions as an ion channel, mediating chloride-dependent currents that enhance cell apoptosis and the release of progeny virions. This ion channel activity of 2B is an important factor in the pathogenesis of the virus and its ability to spread within the host [134]. The multifunction of 2C in these processes is indicative of its integral role in the viral replication machinery [135]. The precursor 2BC from EV-A71 has the ability to bind to SNARE proteins, specifically STX17 and synaptosome-associated protein 29 (SNAP29), which is crucial for the formation of autolysosomes and thus plays a significant role in EV-A71 replication [136] (Table 3).

The viral protein 3A, a membrane-binding protein, interacts with the Golgi protein ACBD3 to recruit phosphatidylinositol-4 kinase IIIβ (PI4KB) to the sites of genome replication, thereby promoting viral RNA replication. This interaction suggests a role for 3A in modulating the host cell membrane dynamics to support viral replication [156]. Viral protein 3B, also known as the viral genome-linked protein (VPg), undergoes uridylation, a process that is crucial for the replication of the genomic RNA. The uridylation of VPg is a key step in the initiation of RNA synthesis, as it allows for the priming of new RNA strands [161]. The precursor 3AB has been established as a nucleic acid chaperone protein with a specific region, 3B plus the last 7 amino acids at the C-terminus of 3A (termed 3B+7), exhibiting RNA chaperone activity [180]. The viral 3C^pro^ is central to the proteolytic processing of the viral polyprotein, particularly in the secondary cleavage of precursor proteins. In addition, 3C^pro^ also interacts with viral RNA and participates in genome replication, highlighting its multifunctional nature in the viral life cycle [162]. The viral 3D^pol^, an RNA-dependent RNA polymerase (RdRp), is primarily responsible for the uridylation of VPg and the replication of genomic RNA [172]. As the main enzymatic driver of RNA synthesis, 3D^pol^ is a critical target for antiviral therapies aimed at inhibiting viral replication (Table 3).

### 4.4. Transition from the Viral Translation to Genomic Replication

During EV-A71 replication, the genomic RNA acts as a template for the synthesis of progeny RNA, guiding the synthesis of polyproteins and the subsequent assembly and release of virions. In vitro studies have shown that cycloheximide, which binds to ribosomes and inhibits eEF2-mediated translocation, blocks eukaryotic protein translation elongation and suppresses viral RNA replication. Conversely, puromycin, a tyrosine-tRNA mimic that is incorporated by the ribosome into the C-terminus of elongating nascent polypeptide chains, can enhance viral RNA replication by freeing ribosomes [181]. The inhibitory effect of RNA-binding ribosomes on RNA replication suggests that translation and viral RNA replication cannot occur concurrently on the same RNA molecule, indicating the presence of a molecular switch that inactivates viral translation and initiates genomic RNA replication.

The conserved 5′-UTR of the viral genome is crucial for both RNA translation and genomic replication. Gamarnik et al. [182] proposed that the interaction of the 3CD protein with the cloverleaf (SL I) at the 5′-terminus of the viral genome favors RNA replication over translation. Additionally, poly(rC)-binding protein 2 (PCBP2) is known to bind to SL IV during translation. The binding affinity of PCBP2 to SL I increases upon the engagement of the newly synthesized 3CD protein with SL I. This interaction leads to the dissociation of PCBP2 from SL IV, facilitating viral RNA replication and suppressing viral translation [183].

PV 3C^pro^ or 3CD^pro^ mediate the cleavage of PCBP1 and PCBP2 during the middle and late phases of viral infection. Once cleaved, PCBP2 loses its ability to bind to SL IV in translation. However, the truncated PCBP2 retains the ability to bind to SL I and promote viral RNA replication. It is believed that PCBP2 plays a role in coordinating the transition from viral translation to RNA replication [184].

## 5. Viral Genome Replications

After the virus enters the cell, the host protein 5′-tyrosyl-DNA phosphodiesterase 2 (TDP2) facilitates the cleavage of the covalent bond between VPg and the 5′-terminus of the genomic RNA, releasing VPg. The resultant VPg-free RNA then serves as a template for the synthesis of negative-strand RNA and for protein translation, thus allowing translation of virus-encoded proteins. However, the removal of VPg is not essential for the viral RNA replication [185]. Modulated by the viral proteins, the host cells are markedly altered to favor the synthesis of viral RNAs. Nonstructural proteins rearrange the intracellular membranes, leading to the formation of specialized vesicles, which are further reshaped and interact with the viral or host proteins, anchoring the viral RNA molecules on their surface and forming a replication complex that initiates the genomic RNA replication. The RNA replication involves numerous interactions between components from the virus (RNA and proteins) and the host (proteins, membranes, and lipids). All +RNA viruses are characterized by the assembly of viral replication complexes (VRCs), which contain both viral and host proteins, on intracellular membranes.

The viral translation and genomic replication are highly conserved across various enteroviruses. The viral 3D^pol^ is a key enzyme in the EV-A71 replication. Following the shut-off of the host cell translation and the establishment of a lipid environment conducive to genome replication, 3D^pol^ initially utilizes the viral RNA as a template to synthesize a negative-strand RNA, which subsequently serves as a template for the amplification of the nascent positive-strand RNA molecules. Progeny RNAs could serve as a template for genome replication, viral protein translation, or be assembled into progeny virions.

During EV-A71 genome replication, the synthesis of nascent strands is directed by 3D^pol^, which extends the ribonucleotide chain from the 5′-terminal to the 3′-terminal through a series of sequential binding, allosteric modulation, and translocation events. Elongation of the progeny RNA is mediated by multiple nucleotide addition cycles (NACs). Termination of elongation occurs upon recognition of a termination signal, leading to the separation and release of the progeny strands from the template. The termination of RNA synthesis in EV-A71 is thought to be ρ-factor dependent. The ρ-factor, a highly conserved termination factor, exhibits ATPase activity upon encountering a C-rich sequence at the 3′-end of the transcript RNA, supplying the energy required for translocation and binding to a specific sequence. This interaction induces conformational changes in the RNA polymerase, halting its activity. The double-stranded RNA is then separated by the ρ-factor, resulting in the detachment of the RNA polymerase and ρ-factor from the template strand and ultimately leading to the termination of genome replication [186,187].

### 5.1. Preparation for Genome Replication

#### 5.1.1. Replicative Organelle Biogenesis and Phospholipid Biosynthesis

The localization and anchoring of 3D^pol^ within intramembrane structures known as Replicative Organelles (ROs) are crucial for EV-A71 genome replication. These restructured membrane compartments encapsulate the viral replication complex, providing a stable microenvironment that facilitates viral replication, establishes appropriate replicative sites, and shields the viral RNA from host immune system detection [188]. The prevailing view is that ROs are derived from the ER and/or Golgi membranes. The formation of ROs requires the participation of viral proteins, including 2BC and 3A, as well as a set of specific host factors [188,189]. However, the detailed composition and molecular mechanisms underlying RO formation remain to be fully elucidated.

ROs exhibit a dynamic structure, initially presenting as single-membrane vesicles during the early stages of infection and later evolving into double-membrane vesicles and multi-compartment liposomes, which encompass multiple bilayer phospholipid membranes. These organelles eventually become diffused throughout the cytoplasm [190,191]. ROs primarily originate from ER and the trans-Golgi network, leading to the disassembly of the Golgi apparatus [192]. Observations have revealed that ROs are capable of concentrating viral RNA, viral proteins, and host proteins necessary for genome replication. They also mediate the precise localization of replication-associated factors and enzymes while concurrently protecting the viral RNA from cellular immune responses [193].

**Figure 3:**
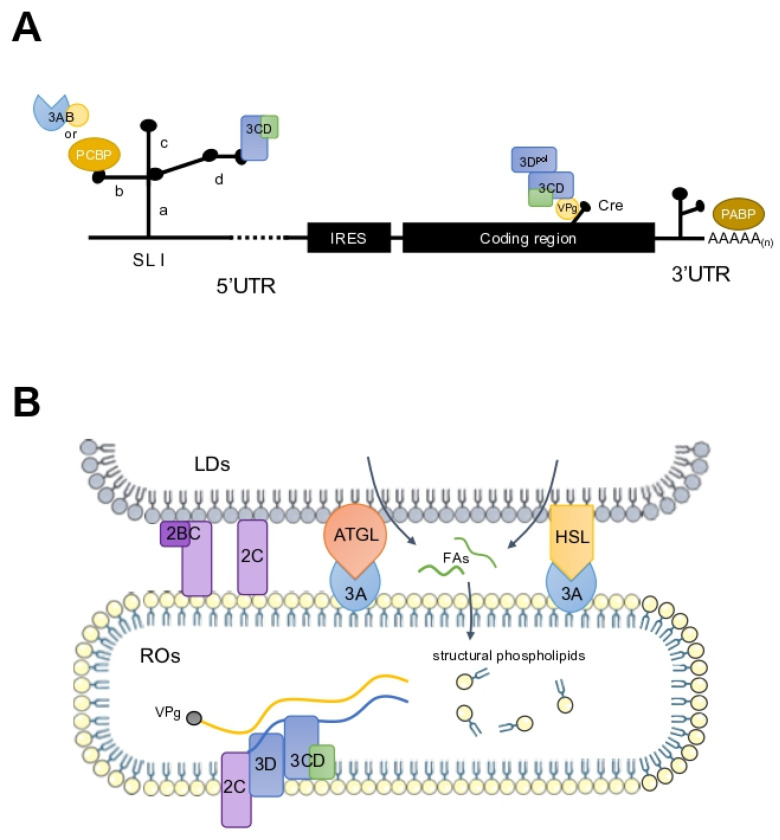
**Schematics of EV-A71 genome replicative-relative complexes, including RNPs and ROs.** (**A**) EV-A71 replicative RNPs. Interactions between viral proteins (3CD, 3AB, 3D^pol^, and VPg) and host cell proteins (PCBP and PABP) with specific cis-regulatory elements of EV-A71 are depicted. These interactions mainly occur at stem-loop I (SL I), which includes four subdomains as alphabetically indicated with a-d, the cis-acting replication element (CRE), and the 3′-poly(A) tract. (**B**) EV-A71 replicative organelles and lipid droplets. Proteins 2C and 2BC facilitate the tethering of lipid droplets (LDs) and replication complexes (RCs). Additionally, 3A and 2BC proteins interact with host lipases ATGL and HSL, promoting the recruitment of lipolytic machinery to the LD-RC interface. These replication complexes are anchored to mature ROs.

EV-A71 can stimulate the biosynthesis of new lipids and the generation of a substantial quantity of ROs, thereby facilitating its own genome replication. This process requires long-chain fatty acids (FAs) from the cell, which serve as substrates for phospholipid synthesis. Studies have shown that the phospholipid synthesis driving RO biogenesis during enterovirus infection is predominantly sustained by FAs derived from lipid droplets (LDs) [188,194] (Figure 3B). LDs, produced in ER, act as key coordinators of lipid metabolism, responsible for the synthesis, storage, and mobilization of neutral lipids. It has been observed that the import of FAs from the extracellular matrix is increased post-infection, a process dependent on acyl-CoA synthetase 3 (ACSL3) [195]. FAs are translocated to LDs to enhance the biosynthesis of phospholipids for ROs [188,194]. The viral 2A^pro^ is deemed necessary for FA uptake, but this process is not dependent on its protease activity, and it requires cofactors to provoke this process. Electron microscopy has revealed distinct membrane contact sites formed between LDs and ROs [188,192]. FAs are then released from LDs by lipases, primarily hormone-sensitive lipase (HSL) and adipose triglyceride lipase (ATGL), which hydrolyze lipid triglycerides. It is proposed that host lipases may be recruited to the LD surface via interactions with several nonstructural proteins upon EV-A71 infection. These lipases are crucial for the release of FAs during EV-A71 infection, affecting RO formation and subsequent RNA replication [188,194]. The released FAs promote the synthesis of structural phospholipids, such as phosphatidylcholine (PC), and accelerate the massive expansion of the membranous network, which is essential for the establishment of ROs and efficient viral replication.

Dynamic mitochondria play a comprehensive role in viral replication, including energy production, apoptosis, innate immunity, cell cycle regulation, and signal transduction. EV-A71 has been shown to suppress type I interferon responses by targeting host mitochondrial adaptor proteins, thereby inducing mitochondria-mediated intrinsic apoptosis [147]. Notably, EV-A71 also triggers the generation of reactive oxygen species within mitochondria, which surprisingly enhances viral genome replication to a certain extent, despite significantly causing mitochondrial dysfunction [196]. The perinuclear aggregation of mitochondria in EV-A71-infected cells was observed by Yang et al. [197]. They further reported a concomitant increase in mitochondrial clustering and viral RNAs, with mitochondria being actively recruited to the viral replication centers. This mitochondrial aggregation serves not only to fulfill the energy requirement but also to modulate the viral replication environment, repurposing mitochondrial proteins and membranes for viral RNA replication. Mitochondrial reorganization in the context of EV-A71 infection is intricately linked to the nonstructural protein 2BC, the microtubule network, the dynein complex, and a low calcium cytoplasmic environment. Further investigations are needed to delineate the molecular mechanisms involved in these interactions.

#### 5.1.2. Creating the Optimal Lipid Microenvironment for Viral RNA Replication

The lipid composition of EV-A71 ROs is characterized by an enrichment of phosphatidylinositol-4 phosphate (PI4P) and cholesterol, which are critical for RO assembly and RNA genome replication [198]. PI4P is synthesized by cellular phosphatidylinositol 4 kinases (PI4Ks), including types II and III, each with A and B subtypes, enabling the generation of localized PI4P in diverse membranes. EV-A71 RNA replication is dependent on PI4KB and PI4P generation at the Golgi membrane [199]. One mechanism by which viral nonstructural proteins recruit PI4KB to ROs involves the Golgi-specific brefeldin A-resistance guanine nucleotide exchange factor 1 (GBF1), which activates Arf1 GTPase to recruit and activate PI4KB [199,200]. A robust interaction between the Golgi adaptor protein ACBD3 and the nonstructural protein 3A has been observed. ACBD3, acting as a collaborative partner for PI4KB, facilitates the recruitment of PI4KB to ROs. Depletion of ACBD3 markedly impedes viral replication [201]. Another PI4KB-binding protein, c10orf76, has also been shown to interact with ACBD3 and is essential for some enterovirus replication [202]. Knockdown of c10orf76 leads to a decrease in PI4P in the Golgi and a translocation of GBF1 [203,204]. Consequently, enteroviruses elevate local PI4P levels in the host mainly through the interplay between the viral 3A and PI4KB, recruiting PI4KB to ROs. Given that 2BC or 3CD of PV can trigger PI4P synthesis, other enteroviruses might similarly modulate the levels of PI4P during viral infections [205,206].

In vitro analysis has demonstrated that the viral 3D^pol^ exhibits a preferential and specific affinity for PI4P lipids [199]. PI4P is capable of directly interacting with viral proteins involved in RNA replication, promoting the association of the replicative complex with membranes or viral RNA. Moreover, PI4KB is implicated in the junction between the 3A and 3B proteins [207], indicating that an appropriate lipid environment is involved in the proteolysis of the viral polyprotein. PI4P also stimulates lipid flux and recruits cholesterol to ROs. It is deduced that cholesterol likely modulates membrane fluidity to participate in viral RNA replication without reliance on its de novo synthesis [208,209]. However, it is not excluded that cholesterol from the original pools likely enhances its acquisition from the plasma membrane through the upregulation of clathrin-mediated endocytosis [209,210] or the lipolysis of lipid droplets [188,208]. Studies suggest that EV-A71 engages in the formation of ROs by utilizing PI4P to transport cholesterol from the ER membrane to the Golgi membrane [205,208,211]. A key molecule in this process is the oxidized low-density lipoprotein-binding protein (OSBP), a lipid transporter that establishes a membrane contact site between the ER and the trans-Golgi, thereby facilitating the exchange of PI4P and cholesterol between the organelles [212].

ROs within host cells present potential therapeutic targets for the inhibition of enterovirus replication. Compounds that target key enzymes involved in lipid metabolism have been shown to effectively suppress enterovirus replication. Notably, the kinase PI4KB is crucial for a multitude of enteroviruses, and various inhibitors targeting this enzyme have demonstrated broad-spectrum antiviral activity in human cells and murine models [199,213]. Similarly, the conserved host factor OSBP is susceptible to inhibition by several antiviral compounds [211,214]. Drug-resistant mutants have revealed that the viral 3A-mediated RO formation can be independent of PI4KB, suggesting that the PI4KB/OSBP pathway may not be necessary for enterovirus replication in vitro [215].

### 5.2. Initiation of EV-A71 Genome Replication

#### 5.2.1. Formation of the Replication Complex

EV-A71 RNA replication is contingent upon the interaction between viral and cellular proteins, culminating in the formation of a replicative complex, termed the ribonucleoprotein complex (RNP), in the presence of an RNA template. The viral 3D^pol^ can replicate any template RNA in vitro when primers are available. However, specific genomic elements within the RNA direct 3D^pol^ to selectively amplify the viral genomic RNA during infection. These elements are situated within the stem-loops of the 5′-UTR, 3′-UTR, cis-regulatory element (CRE), and the 3′-terminal of the negative-strand RNAs. The assembly of these replicative complexes requires template-specific RNA secondary and tertiary structures (Figure 3A). During viral RNA replication, viral and cellular proteins interact with the viral RNA to form a large RNP, which is anchored to virus-induced ROs. This anchoring ensures the availability of necessary proteins and specific RNA templates for efficient replication.

Stem-loop I (SL I) of the 5′-UTR is a crucial regulatory element for both EV-A71 translation and RNA replication. Destabilization of SL I has been shown to impair the synthesis of negative-strand RNA without affecting positive-strand RNA synthesis [216]. Mutational analysis of PV has identified SL I-d as responsible for binding the viral protein 3CD and SL I-b for binding the host protein PCBP2, with these interactions being essential for viral RNA replication [217,218]. The initiation of minus-strand synthesis is believed to occur at the 3′-terminal poly(A), suggesting the presence of long-distance interactions facilitated by RNPs at the termini of the genome. However, the detailed mechanism by which the ternary complex formed by cloverleaf I, 3CD, and PCBP directs RNA synthesis remains unclear. Herold et al. [219] argued that RNP interactions are established at both the viral 5′- and 3′-termini. While PCBP1 is dispensable for this long-range interaction, other cellular proteins like PABP2 could potentially fulfill this role. Since RNA synthesis is unaffected by decreasing the level of PABP1, it is conceivable that PABP1 is not a critical participant in the long-range interaction between the viral 5′- and 3′-termini but is facilitated by viral proteins 3CD and 3AB [220].

The viral 3′-UTR possesses a complex higher-order structure that is essential for RNA replication. Interactions between the 3′-UTR and viral proteins, including the viral 3D^pol^ and the genome-linked protein VPg, are critical for their positioning at the initiation site of minus-strand RNA synthesis. Notably, the complex formed between 3CD and the 3′-UTR in the presence of 3AB is involved in the initiation of negative-strand RNA synthesis [220]. The poly(A) tract in the 3′-UTR is a common feature among picornavirus genomes, although its length varies among different virus species, with a minimum of 12 adenylate residues required for RNA synthesis.

#### 5.2.2. The VPg-pUpU Primers and Initiation of the Viral RNA Replication

The viral protein VPg (3B) is processed by 3D^pol^ and serves as the primer for viral RNA replication, covalently binding to the viral genome to initiate the replicative process. VPg is selectively cleaved from the translational template RNA and remains intact in both the negative-strand RNA and the progeny positive-strand RNA [221]. Distinct replication initiation sites exist for the synthesis of plus-strand and minus-strand RNA, with VPg being predominantly transported to the initiation site by the viral protein 3AB, which is essential for replication initiation [220].

The cis-acting replication element (CRE) is an indispensable RNA structural component for RNA replication, initially identified within the genome of HRV14 [222]. CRE is a conserved stem-loop structure present in the enteroviral genome, characterized by a conserved AAAC sequence. The location of CRE within the viral genomes varies among different virus species; in the case of EV-A71, it seems situated within the 2C-coding region. CRE serves as the template for the uridylation of VPg and participates in the production of primers (VPg-pUpU) for RNA synthesis, a process facilitated by 3D^pol^ and 3CD [138]. After the identification of CRE, it was conventionally considered to be required solely for the synthesis of plus-strand RNA. However, studies on PV observed an inhibition of minus-strand RNA synthesis post-VPg uridylation, suggesting that CRE might also play a role in minus-strand RNA synthesis [223]. Beyond VPg uridylation, CRE also reduces the UTP concentration necessary for the initiation of viral RNA replication [224].

EV-A71 exhibits a disparity in the quantities of plus-strand and minus-strand RNA, with the former being significantly more abundant than the latter, a phenomenon similar to that observed in PV. Consequently, free minus-strand RNA is rarely detectable within host cells as it predominantly binds to the plus-strand RNA, forming either a replicative form (RF) or a replicative intermediate (RI). The RF is a double-stranded RNA arising from the annealing of the full-length plus-strand with its complementary minus-strand, which occurs at a low rate during viral infection. Moreover, RI consists of a full-length minus-strand and multiple plus-strands of various lengths synthesized using the minus-strand as a template [225].

## 6. Virion Assembly and Release

### 6.1. Assembly of the Viral Capsids into Virion

The replicative cycle of EV-A71 culminates in the assembly of infectious progeny viruses, a process well-known to occur in the cytoplasm following viral translation and RNA replication. Initially, 2A^pro^ cleaves the polyprotein at the Y/G site between P1 and P2, resulting in the isolation of P1, which then associates with HSP90. Subsequent hydrolysis mediated by the viral 3CD^pro^ and 3C^pro^ yields VP0, VP1, and VP3 [121]. These cleaved proteins coalesce into 5S protomers, comprising VP0, VP3, and VP1, which serve as the foundational units of the viral capsid and self-polymerize into 14S pentamers (Figure 1). The myristate moiety of the EV-A71 pentamer is recognized as a structural stabilizing factor, with glutathione potentially playing a similar role [226]. A total of 12 pentamers form the 75S hollow viral capsid [(VP0, VP3, VP1)5]12, encapsulating the progeny genomic RNA to create the immature 150S precursor virus. Ultimately, the genomic RNA directs the autohydrolysis of VP0 into VP2 and VP4, yielding the mature virion [(VP4, VP2, VP3, VP1)5]12RNA [63]. It is proposed that multiple sites within the enterovirus genomic RNA have high affinity for the homologous viral capsid proteins, enabling RNA-dependent viral assembly, with pentamers directly binding to and encapsulating the genomic RNA within a protective shell [227,228]. Studies on other picornaviruses suggest that the VP0 cleavage sites guide viral capsid assembly and maturation, although such sites have not been characterized in EV-A71 [229]. Most RNA viruses possess at least one packaging signal that ensures the specificity and accuracy of the viral assembly. However, no evidence delineates the packaging signal for EV-A71. It has been suggested that the specificity of EV-A71 assembly is solely directed by the interaction between the viral 2C and VP3 proteins within the replicative complex, without the need for packaging signals [153].

The assembly of enteroviruses can be impeded by Geldanamycin (GA), a heat shock protein inhibitor with cytotoxic effects, which exerts its activity by targeting the ATP/ADP binding site on the N-terminus of HSP90. Current research about GA mainly focuses on its anti-tumor capabilities [230]. Glutathione is recognized for its multifaceted role in modulating virus assembly, including its ability to interact with capsid proteins and foster the formation of pentameric intermediates. However, there is no compelling evidence to support that EV-A71 relies on glutathione for efficient assembly [231]. Additionally, Vemurafenib, a compound known to specifically inhibit the Raf-MEK-ERK signaling pathway, has been demonstrated to have a modest inhibitory impact on EV-A71 assembly and viral genomic replication [232].

### 6.2. Release of Progeny Virus

It is widely accepted that EV-A71, like other enteroviruses, primarily facilitates the release of progeny viruses through cellular lysis. The cytolytic release mechanism is a fundamental pathway for non-enveloped viruses to release their progeny, mainly accomplished through cell death and lysis. Studies have demonstrated that EV-A71 2A^pro^ and 3C^pro^ are capable of inducing cell death. Specifically, 2A^pro^ can induce the cleavage of eIF4GI, an event that results in rapid cell death by shutting down cell translation, while 3C^pro^ mainly facilitates the release of progeny viruses through the activation of the caspase-dependent apoptotic pathway [233,234].

However, recent studies have shown that enteroviruses can also be released without causing cell death. This non-lytic release is achieved by enhancing autophagy in host cells, leading to the formation of double-membrane autophagosomes that encapsulate virions [235]. The viruses evade degradation by inhibiting the fusion of these specialized autophagosomes with lysosomes. Subsequently, the autophagosomes containing virions fuse with the cell membrane, releasing virion-laden monolayer vesicles into the extracellular environment [22]. These monolayer vesicles are enriched with phosphatidylserine, which triggers their uptake by adjacent cells through phosphatidylserine receptors, thereby initiating subsequent infection cycles [235]. Despite this, enteroviruses within these vesicles still require authentic viral receptors for new infections. The non-lytic release is believed to increase viral infectivity and represents a strategy for viruses to evade neutralizing antibodies [236]. However, the detailed mechanisms and implications for lytic-capable viruses accommodating the non-lytic release mode as well as their roles in viral transmission and pathogenesis warrant further investigation.

A small upstream protein (UP), encoded from the uORF in the viral genome of enterovirus species A-C, is beneficial to viral propagation within intestinal cells, mainly by conferring progeny virus release [66]. Studies have shown the presence of a transmembrane region (TM) within the UP, enabling it to bind to the cell membrane and participate in the egress of the virus from the cells [66]. However, there is no evidence to support its involvement in non-lytic release. UP likely functions as a membrane modulator, enhancing the release of viral particles from specialized vesicles within the cells.

In a colonic organoids model, it has been demonstrated that EV-A71-infected cells can be mechanically extruded into the apical extracellular environment with viral infectivity [237]. This extrusion is contingent upon pressure sensing mediated by mechanosensitive ion channels rather than cell apoptosis. When the pressure sensing capability is mitigated, infected cells are prone to releasing substantial free viruses instead of being mechanically extruded [237]. The findings suggest that the mechanical extrusion of infected cells offers some protection for the infected organs; however, it simultaneously facilitates viral release and transmission in different organs. This mechanism of cell extrusion may play a role in both host defense and viral transmission, highlighting the complex interplay between the virus and its host.

## 7. Perspectives

EV-A71 infection is primarily linked to mild HFMD but also poses a significant risk as a potential cause of severe neurological complications, particularly threatening infants and young children. This review synthesizes the current understanding of the EV-A71 replicative cycle, drawing extensively on research from other enteroviruses, including PV. While these studies provide a valuable framework for understanding EV-A71 replication, they also highlight several unresolved questions about the molecular events within the viral life cycle. Future research should focus on clarifying the viral tropism, the early molecular events post viral entry, the specific locations of viral RNA translation and replication, and the origins and functions of ROs. Moreover, understanding the mechanisms of viral RNA encapsidation, the involvement of viral proteins and host factors in this process, and the role of virus-laden extracellular vesicles in progeny virus release are critical questions within the EV-A71 replication paradigm. Further in-depth investigations into the viral replicative cycle are expected to reveal comprehensive alterations in host cell biology, regulatory networks, and viral pathogenesis. A profound understanding of virus replication is anticipated to significantly advance the development of antivirals and vaccines, thereby aiding in the control of a range of enterovirus infections, including EV-A71.

## Figures and Tables

**Table 1:** ITAFs involved in EV-A71 IRES-mediated cap-independent translation.

Regulatory Activity	Abbreviation	Full Name	Target Sites or Potential Mechanisms	References
Inhibitory	hnRNP D, AUF1	Heterogeneous nuclear, AU-rich element binding factor 1 ribonucleoprotein D	SL II; compete with ribosome for IRES binding sites	[92]
	APOBEC3G, A3G	Apolipoprotein B mRNA-editing enzyme catalytic polypeptide 3 protein G	SL I, SL II; compete with PCBP1 for IRES binding sites	[93]
	vsRNA 1	Virus-derived small RNA 1	SL II; processed by Dicer	[94]
	SIRT1	Silent mating type information regulation 2 homolog 1	SLI, II, III, and V	[95]
	G3BP1	Ras-GAP SH3-binding protein 1	Unknown	[96]
	FBP2, KHSRP	Far upstream element binding protein 2, KH-type splicing regulatory protein	SL I-II, SL II-III, SL V-VI, and 5′-linker region; competes with FUBP1 for IRES	[83,97]
*Inhibitory*	*FBP2_190-711_*	*Cleaved FBP2 (N-terminus)*	5′-UTR	
*Stimulatory*	*FBP2_1-503_*	*Cleaved FBP2 (C-terminus)*	5′-UTR	
Stimulatory	Hsp27	Heat shock protein 27	Enhance 2A^pro^ functions	[84]
	Hsc70, HSPA8	Heat shock cognate protein 70	Non-IRES regions of genomic RNA; promote the cleave of eIF4G by 2A^pro^	[85,98]
	DDX3	The DEAD-Box RNA Helicase	5′-UTR; promote eIF4G cleavage by 2A^pro^ and ribosome entry	[86]
	PCBP1	Poly(C)-binding protein 1	SLI and IV	[99]
	PCBP2	Poly(C) binding protein 2	SL IV	[68]
	FUBP1, FBP1	Far upstream element binding protein 1	5′-linker region; cleaved by 2A^pro^ and acts additively with full-length FBP1	[82]
	FUBP3	Far upstream element-binding protein 3	5′-UTR	[100]
	*FBP1_1-371_*	*Cleaved FBP1*	5′-linker region	
	HuR, ELAVL1	Human antigen R, ELAV-like RNA-binding protein 1	SL II; affect vsRNA1	[101]
	AGO2	Argonaute 2	SL II; affect vsRNA1	[101]
	KHDRBS1, Sam68	KH RNA binding domain containing, signal transduction associated 1	SL IV and V; interacts with PCBP2 and PABP	[90]
	hnRNP K	Heterogeneous nuclear ribonucleoprotein K	SL II, IV; interference with PTBP recognition, antagonistic to 3C^pro^	[102,103,104]
	hnRNP A1	Heterogeneous nuclear ribonucleoprotein A1	SL II, VI; methylated by PRMTs, enhanced interaction with IRES	[105,106]
	PTBP1	Polypyrimidine tract binding protein 1	SL VI	[107]
	MOV10	Moloney leukemia virus 10 (C-terminus domain)	SL I, IRES	[108]
	GADD34, PPP1R15A	Growth arrest and DNA damage-inducible protein 34	5′-UTR	[109]
	HNRNP F	Heterogeneous nuclear ribonucleoprotein F	5′-UTR	[110]
	HNRNP H	Heterogeneous nuclear ribonucleoprotein H	5′-UTR	[110]
	Staufen1	Staufen homolog 1	5′-UTR	[111]
	EGR1	Early growth response-1	SLI and IV	[91]

**Table 2:** Structural proteins of EV-A71 *.

Proteins	Sizes	Main Functions	Potentially Involved Pathways or Mechanisms	References
VP1	297 aa	Receptor binding epitopes	Binds to SCARB2, PSGL-1, and other receptors	[39,46,51,52]
		Stabilizes virions; mediates uncoating, genome release	Contains the canyon, pocket factor, and their discharge	[57]
		Increases cell tropism and host adaptation	Non-conserved mutations alter viral immunogenicity	[124]
		Assembly and maturation of progeny virions	Conserved Ala at 107 regulates VP0 precursor cleavage	[125]
		Neurovirulence determinant	Regulating cell autophagy by mTOR	[126]
VP2	254 aa	Highly conserved antigenic determinants	Amino acids residues 28 and 142–146 (EDSHP)	[123,127]
VP3	245 aa	Epitopes for neutralizing antibodies	Highly conserved “Knob” region of VP3 protein	[128,129]
VP4	69 aa	Stabilizes the virion and involved in uncoating	Ligation with genomic RNA	[63,130]
		Enhance viral infectivity	Myristoylation at the N-terminal of VP4	[131]
		Potential antibody target	VP4N20 (the first 20 amino acids at the N-terminal of VP4 in the EV71 genotype C4)	[132]

* In addition to being capsid proteins, other well-known functions are listed as well.

**Table 3:** Non-structural proteins of EV-A71.

Proteins	Sizes	Main Functions	Potentially Involved Pathways or Mechanisms	References
2A^pro^	∼150 aa	Translation initiation, polyprotein processing	Autohydrolysis and proteolytic activity, cleave eIF4G1	[89]
		Promote RNA replication	Inhibit synthesis of P-body	[137]
		Facilitate virus replication	Positively mediate ERK signaling	[133]
		Induce cell apoptosis	Cleave eIF4G1	[138,139]
		Inhibit nuclear transport	Cleave Nup62	[140]
		Immune evasion	Activation of NLRP3 inflammasome	[141]
			Cleave IFNAR1 (JAK/STAT signaling)	[142]
			Cleave MAVS, MDA5, lower IFN-α/β	[143]
			Block IFN-mediated Jak/STAT signaling by decreasing IFNAR1 levels	[144]
			Attenuated IFN-γ signaling by reducing the serine phosphorylation of STAT1	[145]
2B	∼100 aa	Progeny virus release	Forming ion channels, mediate chloride-dependent current	[134]
		Facilitate virus replication	Interact with VDAC3, enhance ROS production of mitochondria	[146]
		Induce cell apoptosis	Activation of pro-apoptotic protein Bax, up regulate Ca^2+^	[147,148]
		Immune evasion	Inhibit JAK/STAT signaling	[149]
2C	329 aa	Promote RNA replication	NTPase, Format and directing replication complexes to cell membranes.	[135,150]
		Virus uncoating	Revertant 2C-31R1 with secondary point mutation was defective in virion uncoating	[151]
		Cellular membrane rearrangement	Formation of extensive tubular membrane structures	[152]
		RNA encapsidation	Domain near to C-terminal with specific affinity to capsid protein(s), especially VP3	[153,154]
		Immune evasion	Bond to p65, inhibit p65/p50 aggregate	[150]
			Inhibit IKKβ phosphorylation	[155]
3A	86 aa	Promote RNA replication	Interact with ACBD3, recruit PI4KB to replication complex with SCAMP3.	[156,157]
		Facilitate virus replication	Promote exosome biogenesis, interact with PRSS3	[158,159]
		Immune evasion	Induce expression of G3BP1, inhibit RLH signaling	[96]
			Interact with ATP1B3, up-regulate IFN-I	[160]
3B	∼22 aa	Promote RNA replication	Uridylation for the primer of RNA synthesis	[161]
3C^pro^	184 aa	Polyprotein processing	Proteolytic activity	[162]
		Translation initiation	Cleave CstF-64, block host mRNA polyadenylation, promote translation initiation	[163]
			Cleave Stau2 into 508–570 aa that promotes EV-A71 replication	[111]
		Promote RNA replication	RNA-binding activity	[162]
		Immune evasion	Cleave TAK1/ TAB1 / TAB2/ TAB3, inhibit expression of cytokine	[164]
			Cleave RIG-I, MAVS, IRF7, TRIF, and ZMYM2; downregulated miR-526a	[143]
			Cleave PML IV, inhibit formation of PML nucleosomes	[165]
			Cleave host antiviral factor OAS3	[166]
			Decrease interaction of mutant type 3C^pro^ with TRIM21, evade immune recognition	[167]
			Cleave the immune-associated protein TRAF3IP3	[168]
		Inhibit pyroptosis	Cleave GSDMD	[169]
		Induce cell apoptosis	Cleave hnRNP A1, activation of CAS apoptotic protease	[170]
		Mediate neurological symptoms	Cleave TDP-43 and transfer to cytoplasm	[171]
3D^pol^	∼462 aa	Promote RNA replication	Mn^2+^ dependent tRNA polymerase, mediate uridylation of VPg Modified by SUMO1, assist in replicative organelle assembly	[172,173]
			Interacts with 5′-UTR that associated RNA replication	[174]
		Facilitate virus replication	Interact with BECN1	[175]
		Induce S-phase block	Promote Cyclin E1 transcription, regulate CDK2 expression	[176]
		Trigger inflammatory response	Bind to NLRP3, promote inflammasome formation, facilitate IL-1β maturation	[177]
		Immune evasion	Block STAT1 translocation, down-regulate IFN-γ	[178]
			Suppress expression of PGAM5, IFN-β	[179]

## Data Availability

Data sharing is not applicable.
